# Supplementary material for: Comparison of Chromatic and Spectrophotometric Properties of White and Red Wines Produced in Galicia (Northwest Spain) by Applying PCA
Source: Molecules. 2022 Oct 18;27(20):7000. doi: 10.3390/molecules27207000 (PMC9609369; doi:10.3390/molecules27207000)
Supplement: Supplementary file 1 [file molecules-27-07000-s001.zip › molecules-1955896-supplementary.pdf]

Table S1. Some chromatic characteristics of Galician red wines.

Wine codes	Color			CIELab	
	% yellow	% red	% blue	a	b
R1	34.29 vwx	54.34 ab	11.38 r	8.97 o	5.44 ijklmn
R2	33.60 x	54.46 ab	11.94 qr	-3.13 p	0.52 p
R3	51.10 efg	11.09 s	37.81 ab	20.42 hijkl	4.17 ijklmnop
R4	53.52 abcd	10.46 s	36.02 abcd	26.68 abcdef	5.82 ghijklmn
R5	37.29 tu	38.59 jklm	24.12 e	16.19 lm	2.71 mnop
R6	41.28 qrs	42.79 fg	15.93 jklmn	22.49 defghij	4.51 ijklmno
R7	38.19 tu	44.72 ef	17.08 hijkl	22.7 defghij	4.10 ijklmnop
R8	33.98 wx	53.78 ab	12.25 qr	0.26 p	2.35 nop
R9	51.96 def	11.99 s	36.05 abcd	20.02 ijkl	3.41 klmnop
R10	53.88 abcd	10.34 s	35.78 abcd	29.19 ab	6.38 ghijklm
R11	39.79 rst	44.58 ef	15.62 jklmno	24.03 bcdefghi	4.92 ijklmn
R12	33.71 x	53.94 ab	12.35 pqr	7.48 o	4.49 ijklmno
R13	51.77 def	11.36 s	36.87 abc	29.83 ab	6.68 ghijkl
R14	55.04 a	11.19 s	33.77 d	28.47 abc	6.06 ghijklmn
R15	38.64 stu	39.62 hijkl	21.74 ef	23.64 cdefghij	5.65 hijklmn
R16	39.63 rst	46.40 e	13.97 mnopqr	26.79 abcdef	5.71 hijklmn
R17	33.52 x	54.26 ab	12.22 qr	7.57 o	5.02 ijklmn
R18	50.79 efg	11.59 s	37.62 ab	31.00 a	7.61 ghi
R19	53.13 abcde	10.95 s	35.92 abcd	28.90 abc	6.47 ghijklm
R20	37.69 tu	41.23 ghi	21.07 f	25.95 abcdefg	5.26 ijklmn
R21	34.07 wx	54.57 a	11.36 r	7.97 o	4.71 ijklmn
R22	52.02 cdef	10.72 s	37.26 ab	27.05 abcdef	5.56 ijklmn
R23	54.02 abcd	10.73 s	35.25 bcd	30.48 a	7.02 ghijkl
R24	38.88 stu	41.11 ghij	20.01 fg	24.23 bcdefghi	5.17 ijklmn
R25	36.67 uvw	41.99 fgh	21.34 f	24.18 bcdefghi	4.58 ijklmno
R26	51.99 cdef	10.69 s	7.46 s	27.37 abcde	6.39 ghijkl
R27	53.04 abcde	10.41 s	36.56 abc	18.06 jkl	3.30 op
R28	54.86 ab	10.82 s	34.32 cd	28.65 abc	6.66 ghijkl
R29	38.88 stu	41.11 ghij	20.01 fg	7.37 o	0.77 op
R30	36.49 uvw	42.18 fgh	21.33 f	21.54 efghijkl	3.67 ijklmnop
R31	53.20 abcde	10.41 s	7.28 s	29.29 ab	7.14 ghijk
R32	52.26 bcdef	11.26 s	36.48 abcd	27.02 abcdef	5.79 ghijklmn
R33	49.83 fgh	11.98 s	38.20 a	27.96 abcd	7.85 ghi
R34	N.A. x	N.A. t	N.A. t	29.12 abc	9.55 g
R35	38.36 tu	39.60 hijkl	22.04 ef	29.41 ab	9.41 gh
R36	47.75 hijk	37.83 lmno	14.42 lmnopq	22.67 defghij	26.80 c
R37	45.16 klmn	37.72 lmno	17.13 hijkl	25.75 abcdefgh	22.28 cd
R38	51.63 defg	32.24 qr	16.12 jklmn	16.94 klm	30.69 b
R39	47.95 hij	35.03 opq	17.03 hijkl	21.88 efghij	24.25 cd
R40	46.15 jklm	40.87 ghijk	12.98 opqr	22.70 defghij	6.75 ghijkl
R41	43.70 mnopq	38.17 klmn	18.13 ghij	20.59 ghijkl	5.45 ijklmn
R42	46.91 ijkl	35.53 nop	17.56 ghijk	29.20 ab	20.67 de
R43	44.85 lmno	41.23 ghi	13.93 nopqr	18.25 jkl	4.32 ijklmno
R44	51.51 defg	34.81 pq	13.68 nopqr	25.73 abcdefgh	27.33 bc
R45	42.14 opqr	38.87 ijklm	18.99 ghi	29.36 ab	17.58 ef
R46	44.27 lmnop	41.20 ghi	14.53 lmnopq	29.50 ab	17.59 ef

R47	32.84 x	52.14 bc	15.02 klmnop	10.51 no	0.40 p
R48	32.74 x	49.92 cd	17.35 ghijk	11.96 mno	0.41 p
R49	37.47 tu	46.92 e	15.60 jklmno	31.02 a	6.74 ghijkl
R50	36.81 uv	47.23 de	15.96 jklmn	28.85 abc	5.92 ghijklmn
R51	43.34 nopq	42.53 fg	14.14 mnopq	29.21 ab	15.20 f
R52	49.03 ghi	36.53 mnop	14.44 lmnopq	27.08 abcde	15.08 f
R53	48.06 hij	37.75 lmno	14.19 mnopq	26.29 abcdef	17.39 ef
R54	54.37 abc	31.98 r	13.65 nopqr	15.17 mn	38.16 a
R55	46.94 ijkl	39.54 hijkl	13.51 nopqr	27.94 abcd	15.08 f
R56	41.74 pqr	38.64 ijklm	19.62 fgh	24.37 bcdefghi	6.79 ghijkl
R57	45.53 jklmn	37.76 lmno	16.70 ijklm	21.51 fghijkl	7.25 ghij
R58	46.33 ijklm	40.14 ghijkl	13.53 nopqr	26.31 abcdef	17.13 ef

N.A.: not available. Data values in a column with different lowercase letters are statically different ($p \leq 0.05$)

Table S2. Some chromatic characteristics of Galician white wines.

Wine codes	Color			CIELab	
	% yellow	% red	% blue	a	b
W1	49.55 [mn]	27.16 [efghijk]	23.28 [b]	1.75 [abcd]	7.54 [i]
W2	51.35 [jklm]	26.73 [ghijkl]	21.92 [bc]	1.81 [abc]	7.39 [i]
W3	49.56 [mn]	29.17 [bcdef]	21.27 [bc]	1.77 [abcd]	7.93 [i]
W4	52.07 [ijkl]	27.49 [defghij]	20.44 [cd]	1.57 [abcd]	7.45 [i]
W5	51.04 [jklm]	27.46 [defghij]	21.50 [bc]	1.86 [abc]	7.79 [i]
W6	50.76 [klm]	27.36 [defghij]	21.88 [bc]	1.69 [abcd]	7.29 [i]
W7	51.31 [jklm]	27.41 [defghij]	21.28 [bc]	1.66 [abcd]	7.72 [i]
W8	50.53 [klm]	28.57 [bcdefgh]	20.90 [cd]	1.81 [abc]	8.04 [i]
W9	50.74 [klm]	27.30 [defghijk]	21.96 [bc]	2.07 [ab]	7.93 [i]
W10	46.89 [o]	29.66 [abcd]	23.45 [b]	1.55 [abcd]	7.98 [i]
W11	49.71 [mn]	29.36 [abcde]	20.93 [cd]	1.62 [abcd]	8.37 [i]
W12	52.19 [ijkl]	28.81 [bcdefg]	19.00 [cde]	1.69 [abcd]	8.61 [i]
W13	50.12 [lmn]	29.30 [abcdef]	20.58 [cd]	1.68 [abcd]	8.47 [i]
W14	48.20 [no]	30.15 [abc]	21.65 [bc]	1.44 [abcde]	6.40 [i]
W15	47.88 [no]	30.42 [ab]	21.69 [bc]	1.63 [abcd]	7.26 [i]
W16	47.88 [no]	30.96 [a]	21.16 [bcd]	1.54 [abcd]	7.38 [i]
W17	49.26 [mn]	29.89 [abc]	20.84 [cd]	1.60 [abcd]	7.88 [i]
W18	61.17 [cde]	27.39 [defghij]	11.44 [kl]	3.02 [a]	28.31 [a]
W19	62.61 [bcde]	24.59 [lmnopq]	12.80 [ijk]	-0.13 [defg]	3.26 [j]
W20	61.24 [cde]	24.92 [klmnopq]	13.84 [ghij]	0.21 [bcdefg]	17.44 [f]
W21	63.57 [ab]	23.90 [nopq]	12.53 [ijk]	0.50 [bcdefg]	18.82 [ef]
W22	62.95 [bcd]	24.32 [mnopq]	12.72 [ijk]	0.12 [cdefg]	17.19 [f]
W23	61.69 [bcde]	26.20 [hijklmn]	12.11 [jkl]	0.38 [bcdefg]	17.18 [f]
W24	60.62 [ef]	27.97 [cdefghi]	11.41 [kl]	1.39 [abcdef]	23.95 [bc]
W25	58.51 [fg]	25.45 [ijklmnop]	16.03 [efg]	0.29 [bcdefg]	13.72 [g]
W26	57.92 [g]	27.78 [cdefghi]	14.30 [fghij]	1.11 [bcdef]	19.81 [de]
W27	61.02 [cde]	26.42 [hijklm]	12.58 [ijk]	0.48 [bcdefg]	17.46 [f]
W28	62.86 [bcd]	25.22 [jklmnop]	11.91 [kl]	0.48 [bcdefg]	18.38 [f]
W29	58.32 [fg]	27.23 [efghijk]	14.45 [fghi]	1.10 [bcdefg]	21.03 [de]
W30	42.11 [p]	31.68 [a]	26.21 [a]	-0.80 [g]	10.39 [h]
W31	53.75 [hi]	30.01 [abc]	16.23 [ef]	0.58 [bcdefg]	21.53 [cd]
W32	59.72 [efg]	28.30 [bcdefgh]	11.98 [kl]	1.88 [abc]	25.39 [b]
W33	60.90 [de]	23.68 [opq]	15.42 [efgh]	-0.48 [fg]	16.22 [fg]
W34	63.84 [ab]	22.75 [q]	13.41 [hijk]	-0.44 [efg]	16.53 [f]
W35	54.15 [hi]	28.34 [bcdefgh]	17.51 [e]	N.A. [i]	N.A. [l]
W36	65.34 [a]	24.68 [lmnopq]	9.979 [l]	0.38 [bcdefg]	21.62 [cd]
W37	52.72 [ijk]	26.57 [ghijklm]	20.70 [cd]	-0.46 [efg]	18.72 [ef]
W38	53.21 [hij]	26.34 [hijklm]	20.45 [cd]	0.17 [cdefg]	-3.85 [k]
W39	55.31 [h]	25.76 [ijklmno]	18.93 [de]	-1.82 [h]	10.14 [hi]
W40	53.25 [hij]	26.93 [fghijkl]	19.82 [cd]	-0.02 [cdefg]	2.09 [j]
W41	63.32 [abc]	23.29 [pq]	13.40 [hijk]	-0.45 [efg]	17.47 [f]

N.A.: not available. Data values in a column with different lowercase letters are statically different ($p \leq 0.05$)
